# *Bartonella henselae* and *B. koehlerae* DNA in Birds

**DOI:** 10.3201/eid2003.130563

**Published:** 2014-03

**Authors:** Patricia E. Mascarelli, Maggie McQuillan, Craig A. Harms, Ronald V. Harms, Edward B. Breitschwerdt

**Affiliations:** North Carolina State University, Raleigh, North Carolina, USA (P.E. Mascarelli, M. McQuillan, C.A. Harms, E.B. Breitschwerdt);; North Carolina State University, Morehead City, North Carolina, USA (M. McQuillan, C.A. Harms, R.V. Harms)

**Keywords:** Bartonella henselae, Bartonella koehlerae, wild birds, ITS DNA, intergenic spacer region DNA, vector-borne, zoonotic, bacterial disease, bacteria, zoonoses

**To the Editor**: Bartonellosis, a globally emerging vector-borne zoonotic bacterial disease, is caused by hemotropic, gram-negative, aerobic, facultative intracellular *Bartonella* spp. (*1*). Of the 30 *Bartonella* species/subspecies*,* 17 have been associated with human infections (*2*,*3*). Each species has a reservoir host(s), within which the bacteria can cause intraerythrocytic bacteremia with few or no clinical signs of illness (*1*,*3*); the bacteria are transmitted by hematophagous arthropod vectors (*1*). Various *Bartonella* spp. have been identified in domestic and wild animals, including canids, deer, cattle, rodents, and marine mammals (*1*,*4*). *Bartonella* DNA from the blood of loggerhead sea turtles (*Caretta caretta*) has been PCR amplified and sequenced (*5*); the fact that *Bartonella* DNA was found suggests the possibility that persistent blood-borne infection can occur in nonmammals and that the host range for *Bartonella* spp. may be larger than anticipated.

Growing evidence suggests that wild birds play key roles in the maintenance and movement of zoonotic pathogens such as tick-borne encephalitis virus and *Borrelia* and *Rickettsia* spp. (*6*–*9*). *Bartonella grahamii* DNA was amplified from a bird tick in Korea (*10*). The substantial mobility, broad distribution, and migrations of birds make them ideal reservoir hosts for dispersal of infectious agents. To investigate whether birds might be a reservoir for *Bartonella* spp., we screened 86 birds for the presence of *Bartonella* spp. DNA.

The primary study site was a residential backyard in Morehead City, North Carolina, USA (34°43.722′N, 76°43.915′W). Of the 86 birds screened, 78 (16 species) were captured by mist net during March 2010–June 2012 and 8 (3 species) were injured birds that were to be euthanized (Table). Each bird was examined for external abnormalities and ectoparasites, weighed, measured, and tagged with a US Geological Survey–numbered band. A blood sample (0.10–0.25 mL) was collected from each bird by using a 1-mL insulin syringe with a 28-gauge × 1.27-cm needle. Blood remaining after preparation of blood smears was added to an EDTA tube and frozen (−80°C) until processed. Blood smears were examined for hemoparasites. Research was conducted under required state and federal bird banding permits and with the approval of the North Carolina State University Institutional Animal Care and Use Committee.

**Table:** *Bartonella* species detected in birds*

Bird common name	Bird species	No. birds positive/no. total	*Bartonella* sp.
House sparrow	*Passer domesticus*	0/28	
Boat-tailed grackle	*Quiscalus major*	0/15	
Mourning dove	*Zenaida macroura*	0/12	
Herring gull†	*Larus argentatus*	0/6	
House finch	*Carpodacus mexicanus*	0/5	
Blue jay	*C*y*anocitta cristata*	0/3	
Song sparrow	*Melospiza melodia*	0/2	
Northern cardinal	*Cardinalis cardinalis*	0/2	
Northern mockingbird	*Mimus polyglottos*	2/2	*B. henselae* SA2
European starling	*Sturnus vulgaris*	0/2	
Red-winged blackbird	*Agelaius phoeniceus*	1/1	*B. henselae* SA2
Brown thrasher	*Toxostoma rufum*	0/1	
Tufted titmouse	*Baeolophus bicolor*	0/1	
Red-bellied woodpecker	*Melanerpes carolinus*	1/1	*B. koehlerae*
Common grackle	*Quiscalus quiscula*	0/1	
Common loon†	*Gavia immer*	1/1	*B. koehlerae*
Red-headed woodpecker	*Melanerpes erythrocephalus*	0/1	
Brown pelican†	*Pelicanus occidentalis*	0/1	
Collared dove	*Streptopelia decaocto*	0/1	

*SA2, San Antonio 2 intergenic spacer region genotype. †Euthanized.

Before DNA was extracted from the samples, 10 μL of blood was diluted in 190 µL of phosphate-buffered saline. DNA was automatically extracted by using a BioRobot Symphony Workstation and MagAttract DNA Blood M96 Kit (QIAGEN, Valencia, CA, USA). *Bartonella* DNA was amplified by using conventional *Bartonella* genus PCR primers targeting the 16S–23S intergenic spacer region: oligonucleotides, 425s (5′-CCG GGG AAG GTT TTC CGG TTT ATCC-3′) and 1,000as (5′-CTG AGC TAC GGC CCC TAA ATC AGG-3′). Amplification was performed in a 25-μL reaction, as described (*3*). All PCR reactions were analyzed by 2% agarose gel electrophoresis. Amplicons were sequenced to identify the *Bartonella* sp. and intergenic spacer region genotype. To compare sequences with those in GenBank, we identified bacterial species and genotypes by using Blast version 2.0 (http://blast.ncbi.nlm.nih.gov/Blast.cgi). DNA extraction and PCR-negative controls remained negative throughout the study.

Results are summarized in the Table. None of the screened birds were anemic, but 5 were PCR positive for *Bartonella* spp. (3 for *B. henselae* and 2 for *B. koehlerae*). *B. henselae* was amplified from 2 Northern Mockingbirds (*Mimus polyglottos*) and 1 Red-winged Blackbird (*Agelaius phoeniceus*) (GenBank accession no. KC814161). The DNA sequences were identical to each other and had 99.6% (456/457 bp) sequence similarity with *B. henselae* San Antonio 2 intergenic spacer region genotype (GenBank accession no. AF369529). *B. koehlerae* was amplified from a Red-bellied Woodpecker (*Melanerpes carolinus*) and a Common Loon (*Gavia immer*) (GenBank accession no. KC814162). The DNA sequences were identical to each other (404/404 bp) and to GenBank sequence AF312490. Lice (Mallophaga order) were found on 5 Boat-tailed Grackles (*Quiscalus major*), but no ectoparasites were observed on *Bartonella* spp.–positive birds. Hemoparasites (*Haemoproteus* and *Plasmodium* spp.) were detected in 7 of 86 birds, indicating exposure to hematophagous ectoparasites, but hemoparasites were not detected in the *Bartonella* spp.–positive birds. No bacteria were visualized in *Bartonella* PCR–positive blood smears.

*Bartonella* spp. are increasingly associated with animal and human illnesses; thus, the identification of reservoirs and increased understanding of *Bartonella* spp. disease ecology are of public health importance. Our finding of 2 pathogenic species not previously reported in birds has expanded the potential sources for zoonotic infection.

There is growing evidence that migratory birds serve as reservoirs and/or mechanical vectors for pathogens such as tick-borne encephalitis virus and *Rickettsia* spp. (*6*–*8*). Birds have been implicated as reservoirs for several *Borrelia* spp. (*9*,*10*) and for possible dispersion of other tick-borne pathogens (e.g., *Anaplasma* and *Bartonella* spp.) (*6*,*10*). Tick transmission of *Bartonella* spp. to birds should be investigated, and additional studies that investigate the reservoir host range of *Bartonella* spp. and the transmission of these bacteria to non–host species will improve epidemiologic understanding of bartonellosis and will identify additional risk factors for *Bartonella* spp. transmission to new hosts, including humans.
